# Severe Microcytic Anemia Caused by Complex Hereditary Spherocytosis and X-Linked Sideroblastic Anemia with Mutations in *SPTB* and *ALAS2* Genes

**DOI:** 10.3390/jcm12051990

**Published:** 2023-03-02

**Authors:** Jianying Zhou, Hang Zhang, Yao Qin, Ting Liu

**Affiliations:** Department of Hematology, Institute of Hematology, West China Hospital of Sichuan University, Chengdu 610041, China

**Keywords:** hereditary spherocytosis, X-linked sideroblastic anemia, next-generation sequencing, complex mutations

## Abstract

We report a case of severe anemia caused by complex hereditary spherocytosis (HS) and X-linked sideroblastic anemia (XLSA) with two mutations in the spectrin beta (*SPTB)* and 5-aminolevulinic acid synthase (*ALAS2*) genes. The proband was a 16-year-old male with severe jaundice and microcytic hypochromic anemia since his childhood. He had more severe anemia requiring erythrocyte transfusion, and had no response to vitamin B_6_ treatment. Next-generation sequencing (NGS) revealed double heterozygous mutations, one in exon 19 (c.3936G > A:p.W1312X) of the *SPTB* gene and another in exon 2 (c.37A > G:p.K13E) of the *ALAS2* gene, and confirmed again by Sanger sequencing. The mutation of *ALAS2* (c.37A > G) is inherited from his asymptomatic heterozygous mother, causing amino acid p.K13E, and the mutation has not yet been reported. The mutation of *SPTB* (c.3936G > A) is a nonsense mutation, leading to a premature termination codon in exon 19, and the mutation in the *SPTB* gene is not found in any of his relatives, which indicates a de novo monoallelic mutation. Conclusions: The double heterozygous mutations in the *SPTB* and *ALAS2* genes lead to the joint occurrence of HS and XLSA in this patient, and are implicated in the more severe clinical phenotypes.

## 1. Background

Hereditary spherocytosis (HS) is a common inherited hemolytic anemia caused by mutations in genes coding erythrocyte membrane proteins, which leads to defects in the membrane proteins, including ankyrin, band 3, β-spectrin, α-spectrin, or protein 4.2. Erythrocyte membrane defects reduce deformability, and the abnormal red blood cells (RBCs) are trapped and destroyed in the spleen, which result in hemolysis, anemia, jaundice, splenomegaly, and gallstones. The clinical severity of the disease depends on the extent of the surface area loss and ranges from asymptomatic forms to severe anemia cases requiring erythrocyte transfusion [1].

X-linked sideroblastic anemia (XLSA) is the most common hereditary sideroblastic anemia and is caused by mutations in the erythroid-specific 5-aminolevulinic acid synthase (ALAS) gene, which is located on chromosome Xp11.21 [2]. ALAS is the first enzyme involved in heme biosynthesis in erythroid cells, and *ALAS2* gene mutation leads to ALAS function deficiency, heme synthesis anomaly, iron overload, and anemia. XLSA occurs preferentially in males and usually presents as asymptomatic or mild microcytic anemia. However, approximately one-quarter of the affected XLSA probands are females, which is mostly associated with a skewed X-chromosome inactivation [3]. Pyridoxal phosphate (PLP) as a cofactor can enhance the activity of ALAS; therefore, vitamin B6 can improve anemia in most XLSA patients [4].

Here, we report a male patient with severe transfusion-dependent anemia. Next-generation sequencing (NGS) revealed double heterozygous mutations in the *SPTB* gene and *ALAS2* gene, characterized with two disease entities, HS and XLSA. In contrast to the human gene bank databases, the *ALAS2* mutation is a novel pathogenic mutation that has not yet been reported. Written informed consent was obtained from the patient and his parents for the publication of this case report and any accompanying images.

## 2. Case Presentation

**Figure 1:**
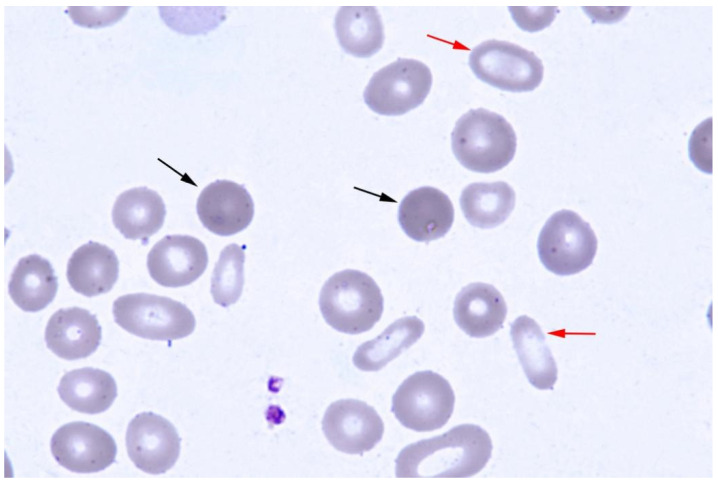
Peripheral blood smear shows anisocytosis, and spherocytes (black arrow) and elliptocytes (red arrow) could be seen easily (Wright’s stain).

A 16-year-old male was admitted to the West China Hospital due to jaundice and anemia that had been present since his childhood. His parents and brother were asymptomatic with normal blood test results. On physical examination, the patient had pale skin, icteric sclera, and hepatosplenomegaly (liver 2 cm below costal; spleen 9 cm below costal). Blood tests showed microcytic hypochromic anemia: hemoglobin 70 g/L (ref value: 130–175 g/L); mean corpuscular volume 77 fL (ref value: 82–100 fL); mean corpuscular hemoglobin concentration 303 g/L (ref value: 316–354 g/L), and the reticulocyte percentage (Ret%) and immature reticulocytes fraction (IRF) were 38.2% (ref value: 0.5–1.5%) and 10.3%, respectively, with a normal white blood cell count (6.14 × 10^9^/L) (ref value: 3.5–9.5 × 10^9^/L) and platelet count (169 × 10^9^/L) (ref value: 100–300 × 10^9^/L). Peripheral blood smear showed erythrocyte morphological abnormalities, including anisocytosis, spherocytes and elliptocytes (shown in Figure 1). His total bilirubin was 83.9 µmol/L (ref value: 5.0–28.0 µmol/L), indirect bilirubin was 78.7 µmol/L (ref value: <20 µmol/L), and lactate dehydrogenase was 319 IU/L (ref value: 120–250 IU/L). For differential diagnosis tests, Coomb’s test was negative, hemoglobin electrophoresis was normal, and no mutation of the α- and β-globin genes was detected by the copy number variation of DNA sequences (CNVplex^®^) and single nucleotide polymorphisms assay (SNapShot). The bone marrow smear showed erythroid hyperplasia with a 44% proportion of total sideroblasts, and the proportion of ring sideroblasts was 30%. Serum ferritin, transferrin, and transferrin receptor (sTfR) were 260 ng/mL (ref value: 24–336 ng/mL), 1.80 g/L (ref value: 2.5–4.3 g/L), and 9.21 mg/L (ref value: 0.76–1.76 mg/L), respectively. All of these results indicated the diagnosis of hemolytic anemia and sideroblastic anemia. 

**Figure 2:**
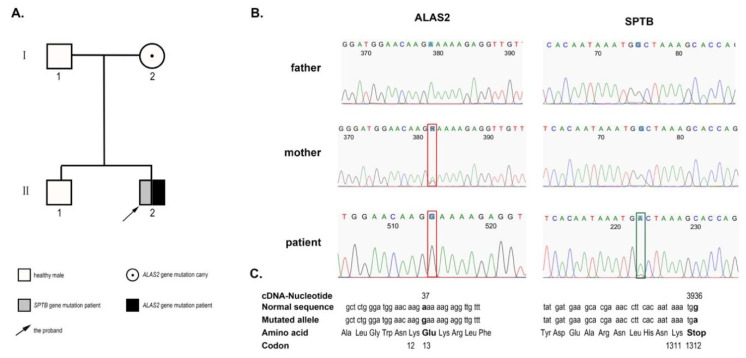
Schematic of the inheritance pattern in the family and the results of NGS. (**A**) Pedigree of the family with hereditary spherocytosis (HS) and X-linked sideroblastic anemia (XLSA). (**B**)The patient harbors a heterozygous mutation c.3936G > A in the *SPTB* gene and a hemizygous mutation c.37A > G in the *ALAS2* gene identified by NGS. The mutation of *ALAS2* is inherited from his asymptomatic heterozygous mother, and the mutation of *SPTB* may be a de novo monoallelic variant. (**C**) *ALAS2* mutation results in amino acid switching (p.K13E), and *SPTB* mutation led to the formation of truncated β-spectrin (p.W1312X).

To confirm the patient’s diagnosis, heparinized anticoagulated blood was obtained from the patient. DNA samples were extracted using a QIAamp DNA Blood MiniKit (Qiagen, Valencia, CA, USA). Then, the DNA sample was subjected to NGS by Kindstar Global Esoteric Test & Services Co., Ltd. (Beijing, China) in a panel of 700 genes for red blood diseases. The primer design, synthesis, and NGS tests were performed in accordance with standard laboratory procedures. The sequences were then aligned to human genome version 19 (HG19) using information from databases, including the NCBI dbSNP, HapMap, and the 1000 Genomes Project human dataset. The sequencing analysis identified double heterozygous mutations, a heterozygous mutation in exon 19 (c.3936G > A:p.W1312X) of the *SPTB* gene, and a hemizygous mutation in exon 2 (c.37A > G:p.K13E) of the *ALAS2* gene. These mutations were confirmed again by Sanger sequencing. Further pedigree investigations were finished for his parents and brother with their informed consent. The results showed that the mutation in exon 19 (c.3936G > A) of the *SPTB* gene identified in the proband was a de novo monoallelic variant because his parents showed a wild type in Sanger sequencing. The mutation in exon 2 (c.37A > G) of the *ALAS2* gene was inherited from his asymptomatic heterozygous mother. The patient’s father and brother were not found to have the two mutations (shown in Figure 2). To our knowledge, the *ALAS2* mutation has not yet been reported. 

The patient was diagnosed with hemolytic anemia caused by hereditary spherocytosis and X-linked sideroblastic anemia. He was treated with folic acid and vitamin B_6_. After 6 months of treatment, he showed little symptomatic improvement, his blood tests indicated severe anemia (Hb 60 g/L; MCV 77.4 fL) with recitulocytosis (12.12%), and his white blood cells and platelet counts were normal.

## 3. Discussion and Conclusions

**Figure 3:**
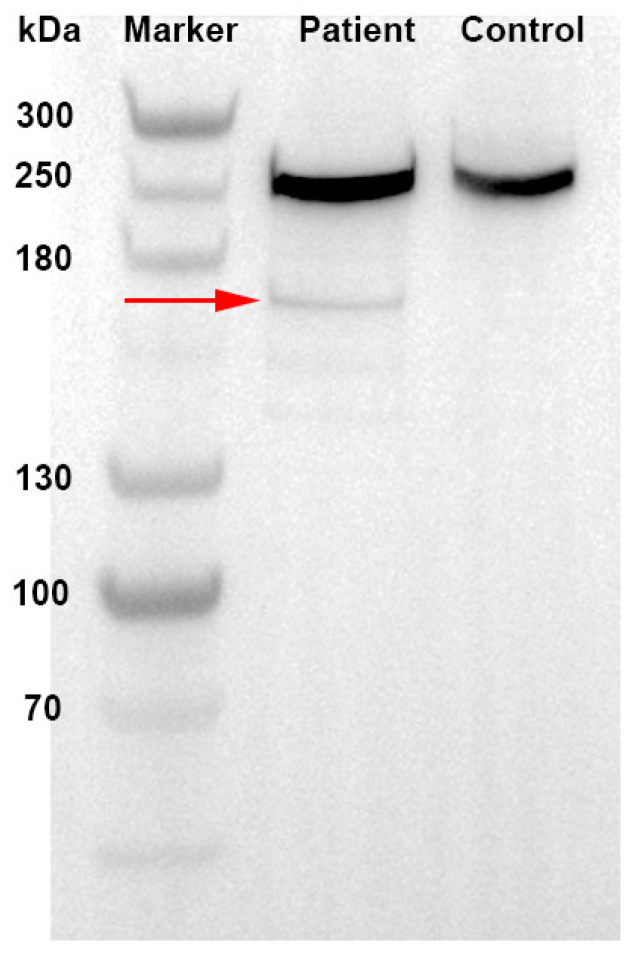
Western blot-based detection of β-spectrin: red arrow showed the truncated β-spectrin.

Spectrins are the major constituent of the erythrocyte cytoskeletal network. Hereditary spherocytosis can be caused by α-spectrin (*SPTA1* gene) or β-spectrin (*SPTB* gene) mutations [5]. In healthy erythroid cells, the production of α-spectrin chains is three-fold to four-fold greater than β-spectrin production. Thus, a mutation of one β-spectrin allele is sufficient to cause spherocytosis, whereas both α-spectrin alleles have to be affected for the disease to arise [6]. The *SPTB* gene codes β-spectrin, along with α-spectrin, to form spectrin heterodimers and finally to form tetramers (α2β2) by a head-to-head connection [7]. The interaction of α2β2 tetramers with other proteins (such as ankyrin, band 3, etc.) forms the biconcave shape of human erythrocytes, which is critical for erythrocyte deformability and stability. The mutation in exon 19 (c.3936G > A) of the *SPTB* gene was found and submitted to the ClinVar database by the researchers from the UOS Fisiopatologia delle Anemie and the Mayo Clinic in 2019, and was published firstly by Fermo E et al. in 2021 [8]. It is a nonsense mutation, which caused tryptophan at position 1312 of β-spectrin chain to change to a termination codon and led to a truncated spectrin-β comprising 1311 amino acids instead of 2328 amino acids in the wild-type counterpart (shown in Figure 3). The c.3936G > A mutation of the patient is located in the *SPTB* anchoring area. The nonsense mutation in the *SPTB* gene may lead to the loss of the ankyrin-binding domain and tetramerization domain of β-spectrin, resulting in the loss of erythrocyte membrane cohesion and lipid vesicle formation. These are responsible for the loss of the cell surface area, increased cell sphericity, and reduced cellular deformability. Abnormal spherocytes are trapped and destroyed in the spleen, resulting in hemolysis, anemia, and jaundice.

The *SPTB* gene mutations were classified into autosomal dominant (AD) inheritance. However, with rare exceptions, mutations of the β-spectrin gene are isolated and might be associated with monoallelic expression, suggesting that null mutations are common [9]. In addition, in our case, the mutation c.3936G > A on exon 19 of *SPTB* led to a termination codon resulting in a truncated β-spectrin, and was only identified in the proband, and not found in any of his family members. That is a de novo monoallelic pathogenic mutation. To date, several de novo *SPTB* mutations have been reported [10,11]. Tole S et al. confirmed nine cases (17%) with de novo monoallelic mutations in 52 patients with *SPTB*-HS [12]. Peng GX et al. reported five de novo monoallelic variants of *SPTB* gene in 14 Chinese patients with *SPTB*-HS [13]. A study from South Korea also identified four de novo *SPTB* monoallelic mutations in 12 *SPTB*-HS patients [14]. These results suggest that the de novo *SPTB* monoallelic mutations are not rare in patients with *SPTB*-HS.

The *ALAS2* gene is located on chromosome X p11.21 and encodes 5-aminolevulinate synthase (ALAS). ALAS is a key rate-controlling enzyme of the heme biosynthetic pathway. In the mitochondria, ALAS catalyzes the formation of 5-aminolevulinate (ALA) from glycine and succinyl CoA. In this reaction, as a cofactor, pyridoxal phosphate (PLP) can enhance the activity of ALAS [15]. Missense mutations in the *ALAS2* gene cause ALAS dysfunction, which hinders the synthesis of ALA and then affects the synthesis of heme, further resulting in iron overload and anemia. Due to an X-linked recessive pattern of inheritance, XLSA typically affects younger males. Approximately two-thirds of cases present in males during early childhood or adolescence and a third in young to middle-aged females [16]. However, as a result of familial-skewed inactivation of the normal X chromosome, females with *ALAS2* mutations may have a late-onset clinical phenotype [3,17]. In our proband, the *ALAS2* gene revealed a missense mutation at c.37A > G of exon 2. The mutation resulted in a change from lysine to glutamate at position 13 of the ALAS2 protein. His mother was an asymptomatic heterozygous carrier.

The human *ALAS2* gene consists of 10 coding exons and a 5′untranslated exon. For the first translated exon (exon 2), the *ALAS2* gene primarily encodes the signal sequence required for targeting the precursor protein to the mitochondrion. The sequence encoded by exons 3 and 4, together with the remainder of the exon 2-encoded sequence that is predicted to not be part of the proteolytically removed mitochondrial targeting sequence, constitutes a 93 amino acid stretch at the N-terminus of the ALAS protein. Exons 5–11 encode the catalytic domain, and most of the identified *ALAS2* point mutations associated with XLSA were located in these exons. Missense mutations of exons 5–11 on *ALAS2* can result in a decreased affinity of PLP with ALAS or can change the stability of the enzyme, so the administration of pyridoxine can restore the enzymatic activity and alleviate anemia [18]. Shoolingin-Jordan et al. found that mutations located in the vicinity of the pyridoxal 5′-phosphate binding site, such as M426V, G291S, T388S and K299Q, exhibit a nearly complete response to pyridoxine therapy [15]. However, XLSA patients with mutations in the promoter region and enhancer region of the first intron, or the creation of premature stop codons were refractory to pyridoxin [19]. In our case, the missense mutation of *ALAS2* was detected in exon 2. This mutation may have little influence on the binding between pyridoxine and ALAS, and on the activity of the catalytic domain, so the therapeutic effect of vitamin B_6_ is not obvious.

In general, regarding the clinical phenotype, patients with β-spectrin deficiency typically have mild to moderate disease manifestation and do not need transfusion. Splenectomy can improve symptoms in most patients, including the alleviation of anemia and hyperbilirubinemia and reduction in the reticulocyte count. XLSA usually presents as asymptomatic or mild microcytic anemia. This proband had more severe anemia requiring erythrocyte transfusion due to the complex hereditary spherocytosis and X-linked sideroblastic anemia with two mutations in the *SPTB* and *ALAS2* genes. After treatment with folic acid and vitamin B_6_, the anemia symptoms of the patient had little improvement, because the mutation of *ALAS2* was not located in exons 5–11 encoding the catalytic domain and not in the vicinity of the pyridoxal 5′-phosphate binding site.

In summary, we reported a severe anemia case caused by the complex hereditary spherocytosis and X-linked sideroblastic anemia with two mutations in the *SPTB* and *ALAS2* genes, a de novo nonsense mutation in the *SPTB* gene (c.3936G > A:p.W1312X) and a missense mutation in the *ALAS2* gene (c.37A > G:p.K13E). The patient did not respond to vitamin B_6_ treatment, possibly because the mutation was not located in the binding site of the encoding catalytic domain of ALAS. The optimal treatment for this patient needs to be pursued.

## Data Availability

The data used to support the findings of this case report are included within the article.

## Abbreviations

AD	autosomal dominant
ALAS	5-aminolevulinic acid synthase
SPTA	spectrin, alpha
HS	hereditary spherocytosis
NGS	Next-generation sequencing
PLP	pyridoxal phosphate
RBCs	red blood cells
SPTB	spectrin, beta
sTfR	serum transferring receptor
XLSA	X-linked sideroblastic anemia
